# Clinical Significance of Non-Invasive Skin Autofluorescence Measurement and AI Applications in Patients with Diabetic Foot Ulcers: A Scoping Review

**DOI:** 10.3390/jpm16060285

**Published:** 2026-05-26

**Authors:** Cosimo Aliani, Piergiorgio Francia, Cosimo Nardi, Alessandra De Bellis, Roberto Anichini, Leonardo Bocchi

**Affiliations:** 1 Department of Information Engineering, University of Florence, Via di Santa Marta 3, 50139 Florence, Italy; piergiorgiofrancia@libero.it (P.F.); leonardo.bocchi@unifi.it (L.B.); 2 Department of Experimental and Clinical Biomedical Science, Viale Giovanni Battista Morgagni 50, 50134 Florence, Italy; cosimo.nardi@unifi.it; 3 Department of Diabetology, S.M. Annunziata Hospital of Bagno a Ripoli, Via Antella 58, 50012 Florence, Italy; alessandra.debellis@uslcentro.toscana.it; 4 Department of Internal Medicine, Diabetes Unit—San Jacopo Hospital of Pistoia, Via Ciliegiole 97, 51100 Pistoia, Italy; roberto.anichini@uslcentro.toscana.it

**Keywords:** diabetic foot ulcer, autofluorescence imaging, wound autofluorescence, artificial intelligence, machine learning, bacterial infection, multispectral imaging

## Abstract

Emerging optical technologies may offer new opportunities for the non-invasive assessment of diabetic foot ulcers (DFUs), but the role of artificial intelligence (AI)-assisted autofluorescence-based approaches remains unclear. This scoping review aimed to map and summarise the published evidence on AI-assisted analysis of autofluorescence/fluorescence-based signals for DFU assessment and management. We searched Scopus, Web of Science, Embase, PubMed, CINAHL, Google Scholar, and the SPIE Digital Library, and also considered conference proceedings. We included English-language studies published between 2010 and October 2025. Of 197 records identified through database searching, 22 full-text articles were assessed for eligibility, and 5 studies met the inclusion criteria. Four studies focused on infection-related applications, specifically bacterial burden detection and Gram-type classification, whereas one study investigated tissue oxygenation estimation using a related optical imaging approach. All included studies were published between 2022 and 2025, were conducted in India, and four of the five evaluated the same device family or related variants. Overall, the evidence base was limited, geographically restricted, and technologically narrow. In addition, reporting of participant characteristics and AI methodology was often incomplete, with several studies relying on embedded proprietary or insufficiently described algorithmic components. Taken together, the available literature supports early proof-of-feasibility in restricted and largely device-specific evaluation settings rather than robust evidence of broad clinical validity, implementation readiness, or routine-care utility. Larger, more diverse, and independently validated studies with standardised acquisition procedures and more transparent AI reporting are needed before these approaches can be meaningfully evaluated for routine DFU care.

## 1. Introduction

Diabetes is one of the most prevalent diseases worldwide. It has been estimated that nearly 600 million adults aged 20–79 years live with diabetes, a number that could rise to over 800 million by 2050 [1,2]. Diabetes is associated with major complications. Among them, diabetic foot ulcers (DFUs) are particularly common and clinically significant because of their impact on morbidity and mortality [3]. The development of foot ulcers can lead to infection, hospitalisation, lower-limb amputation, and death [4]. Up to approximately one-third of people with diabetes may develop a foot ulcer during their lifetime, and DFUs may affect over 6% of the adult population each year worldwide [3,5,6,7]. Consequently, diabetic foot represents a major healthcare concern due to the number of affected patients, the complexity of clinical management, and the associated healthcare and social costs. These factors contribute to substantial disparities in the quality of care across countries and socioeconomic settings [8,9].

The ongoing challenges in preventing and managing diabetic foot highlight the need for continued improvements in patient care. In this context, emerging technologies, including optical systems, have attracted increasing interest [10,11]. Among these, fluorescence-based approaches have been explored for the assessment of people with diabetes and, more specifically, for DFUs complicated by infection [10,12]. Although several studies have suggested a potential role for skin autofluorescence in diabetes-related conditions such as risk stratification and microvascular or macrovascular complications, its role in DFU management remains not yet clearly established.

In this review, we use the umbrella term autofluorescence (AF)-based imaging to refer to endogenous or bacteria-related fluorescence signals acquired without exogenous contrast agents. Where relevant, we distinguish between skin autofluorescence (SAF), measured on intact skin, and wound autofluorescence (WAF), acquired from the wound bed. Although both rely on the excitation of endogenous fluorophores, SAF primarily reflects metabolic and structural tissue properties in intact skin, whereas WAF is more often used to characterise bacterial burden and inflammatory alterations in disrupted tissue environments [13,14]. AF assessment typically involves illumination at specific excitation wavelengths. Upon excitation, endogenous fluorophores in biological tissues (e.g., elastin, keratin, melanin, collagen) [15,16], as well as certain bacterial metabolites, absorb light and subsequently emit fluorescence at longer wavelengths. These emitted signals can then be captured by optical sensors, digitised, stored, and processed to generate images and support quantitative analysis [14,17].

Autofluorescence measurements can produce complex spatial and spectral patterns that may be influenced by multiple biological and technical factors, including tissue composition, perfusion, exudate, wound depth, illumination geometry, and the absorption properties of chromophores such as haemoglobin [18]. These sources of variability are particularly relevant when image-derived signals are translated into clinically interpretable outputs.

In principle, artificial intelligence (AI) may serve as a methodological bridge between optical acquisition and clinically relevant information. It has been proposed for several stages of the workflow, including preprocessing, feature extraction, classification, and quantitative analysis [19]. However, the extent to which AI currently provides robust, reproducible, and clinically transferable advantages in the specific context of DFU autofluorescence assessment remains uncertain.

More broadly, AI-based pipelines—from dataset curation and preprocessing to model development, validation, and deployment—may help address interacting sources of variability, improve robustness to acquisition conditions, and transform raw optical data into quantitative descriptors. Depending on the clinical question, such approaches may also be combined with routinely collected clinical variables to support more standardised assessment and monitoring [20]. However, these potential advantages depend on study design, dataset quality, model transparency, and validation across heterogeneous clinical settings.

Accordingly, the potential contribution of AI in this field should be interpreted cautiously. At present, the literature appears to be emerging and may reflect, at least in part, evaluations of proprietary imaging platforms with embedded algorithmic components rather than a broad and methodologically transparent body of AI research in DFU care [21]. A scoping review is therefore useful to map what has actually been studied, how these systems have been evaluated, and which knowledge gaps remain.

This scoping review aims to map the available evidence on the use of autofluorescence-based or fluorescence-based signals and AI-related analytical approaches in DFU assessment and management, summarise the main characteristics of the identified studies, and highlight current limitations and knowledge gaps. Conceptually, the workflow underlying these technologies spans multiple linked stages: fluorescence-related signals are first generated by endogenous tissue fluorophores and/or bacteria-associated metabolites; these signals are then acquired under specific optical and environmental conditions that may introduce technical variability; the resulting images are processed through steps such as region-of-interest selection, motion correction, feature extraction, and algorithmic inference; finally, the model output is translated into a clinically oriented result, such as estimated bacterial burden, Gram-type suggestion, or tissue oxygenation map. The reliability of the final clinical interpretation therefore depends not only on the algorithm itself, but also on signal biology, acquisition standardisation, and the robustness of the entire analytical pipeline. The review question is as follows: *What studies have investigated AI-assisted analysis of autofluorescence/fluorescence-based signals for diabetic foot ulcer assessment and management, and what are the main characteristics of their populations, acquisition procedures, AI methods, and reported outcomes?*

## 2. Materials and Methods

This scoping review was conducted in accordance with the framework proposed by Arksey and O’Malley [22] and the guidance of the Joanna Briggs Institute (JBI) for scoping reviews [23]. No prospectively registered public protocol was available for this scoping review. Nevertheless, the review was conducted according to a predefined methodological plan established before screening and based on JBI guidance and PRISMA-ScR reporting recommendations [24]. The search strategy adheres to PRISMA guidelines (PRISMA checklist in Supplementary Table S1).

### 2.1. Eligibility Criteria

The review was conducted in five phases [25]:

1. Identifying the research question;
2. Identifying relevant studies;
3. Selecting studies;
4. Charting the data;
5. Collating, summarising, and reporting the results.

To address the research question, we prespecified eligibility criteria. We included original research articles published in peer-reviewed journals and conference proceedings that investigated skin autofluorescence (SAF) signal analysis using artificial intelligence (AI) methods for the non-invasive assessment of DFU status in people with diabetes. We considered all study designs (e.g., randomised controlled trials, cohort, case–control, and cross-sectional studies) to capture the breadth of evidence in this field. Studies had to involve human participants with diabetes; no restrictions were applied regarding age, sex, ethnicity, or comorbidities. The grey literature sources, including book chapters, were not included in the final eligibility criteria.

We focused on the characteristics of dataset construction, data analysis, and AI-based methods, with the aim of assessing whether and how AI-based analysis of autofluorescence signals can support the evaluation of wound status in patients with diabetes. We included studies published between 1 January 2010 and October 2025, and restricted eligibility to articles published in English. The searches were conducted up to 28 October 2025. An updated check of all sources was performed on 26 January 2026 to identify any newly available eligible records.

The lower date limit (2010) was selected because the review focused on contemporary AI-enabled optical imaging applications, which have largely emerged in the last decade. The review was restricted to English-language publications for feasibility reasons; however, this may have introduced language bias and may have led to omission of relevant non-English studies.

We excluded abstract-only records, narrative reviews, systematic reviews, editorials, commentaries, and opinion pieces. Animal studies were also excluded. Studies published before 2010 or in languages other than English were not considered. Additional exclusion criteria were an unclear AI component, defined as no identifiable AI/ML-based analysis beyond generic device output or an insufficient description to establish whether AI materially contributed to signal interpretation, and insufficient reporting of the study population, defined as the inability to confirm inclusion of a diabetic DFU population and/or lack of the minimum participant information needed to assess eligibility.

### 2.2. Information Sources and Search Strategy

We searched the following electronic databases: Scopus, Web of Science, Embase, PubMed, CINAHL (Cumulative Index to Nursing and Allied Health Literature), Google Scholar, and the SPIE Digital Library. Google Scholar was used only as a supplementary search source. A title-restricted strategy was adopted pragmatically to improve feasibility and reduce excessive non-specific retrieval; however, this may have reduced sensitivity and may have led to omission of relevant records.

To identify additional studies not retrieved through database searching, we conducted a supplementary search that included manual screening of reference lists [26]. In particular, backward citation screening of reference lists was the only supplementary search method used. Forward citation tracking, trial registry searches, and hand-searching of specific journals were not performed.

Search terms covered three concept blocks: (i) diabetic foot (e.g., diabetic foot ulcer, lower extremity ulcer, wound healing, neuropathic foot, ischaemic foot, diabetic foot syndrome); (ii) autofluorescence (e.g., autofluorescent, fluorescence, fluorescence imaging, tissue fluorescence, intrinsic fluorescence, skin fluorescence); and (iii) artificial intelligence (e.g., AI, deep learning, neural network, machine learning, genetic algorithm, predictive models, data mining).

Search strings were adapted to the requirements of each database. Full search strategies are reported in Appendix A.

### 2.3. Data Extraction

Two authors (CA and PF) independently screened titles and abstracts to identify eligible records. References were managed in EndNote X9 (Clarivate), and duplicates were removed. Discrepancies were resolved through discussion, and the final set of included studies was compiled in an Excel spreadsheet by one author and verified by a second author. No formal calibration or piloting exercise was performed before screening or data extraction. The same two reviewers independently assessed full-text records for eligibility, and disagreements were resolved by discussion.

We extracted the following information: publication year; study population (including type of diabetes); participant characteristics (sex, BMI, ethnicity, skin characteristics, duration of diabetes, blood glucose and glycated haemoglobin values); therapy; and complications and comorbidities. We also collected details on the autofluorescence device and acquisition procedure; the autofluorescence signal evaluation protocol (including acquisition duration and settings); dataset characteristics and processing; the AI method; and the main outcomes/results. Studies were organised by year of publication, and key information was summarised in tables.

Consistent with the scoping review design, no formal risk-of-bias assessment tool was applied. The purpose of the review was to map the extent, characteristics, and reporting patterns of the available literature rather than to produce a weighted estimate of diagnostic performance. Nevertheless, recurrent methodological limitations of the included studies were systematically noted during data extraction and are discussed narratively in the synthesis.

## 3. Results

### 3.1. Search Results

**Figure 1:**
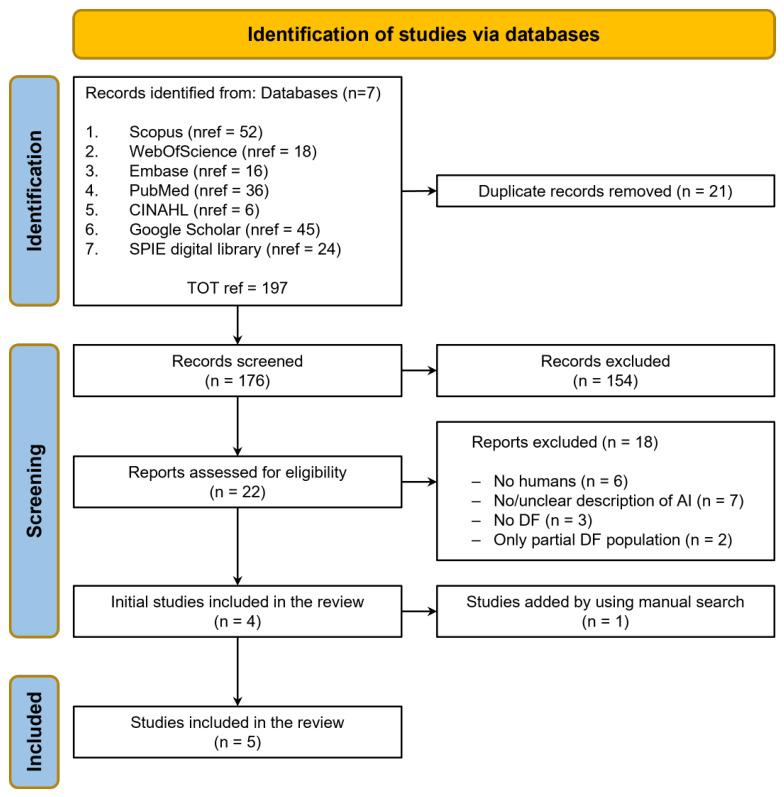
Preferred Reporting Items for Systematic Reviews and Meta-Analyses for Scoping Reviews (PRISMA) flow diagram of the search and study selection process.

A total of 197 records were identified through database searching. After the removal of 21 duplicates, 176 records remained for title and abstract screening. Twenty-two full-text articles were assessed for eligibility. Following full-text assessment, 18 articles were excluded for the following reasons: non-human study (*n* = 6), no AI-based analysis or unclear description of the AI component used to interpret autofluorescence results (*n* = 7), non-diabetic study population (*n* = 3), and insufficient reporting of the study population (*n* = 2). Finally, 4 studies were included from database searching, and 1 additional study was identified through backward citation screening, yielding 5 included studies overall [27,28,29,30,31]. Of the five included studies, four focused on infection detection and Gram-type classification, whereas one addressed tissue oxygenation assessment. The included studies were synthesised in terms of aim, population, protocol/design, analytical methods, AI approach, and autofluorescence-related outcomes. All included studies were published between 2022 and 2025 and were conducted in India. The PRISMA flow diagram is presented in Figure 1, and the included studies are summarised in Table 1.

### 3.2. Scope of the Included Studies and Participant Characteristics

Among the five included studies, four focused on infection-related applications of autofluorescence imaging in DFUs, mainly bacterial burden detection and Gram-type classification [27,28,29,30]. In contrast, Krishnamoorthy et al. [31] described the architecture and operating principles of the “Illuminate OxyView” device and evaluated its performance for tissue oxygenation assessment based on reflectance imaging, including the impact of skin type/melanin on measurements. Because this study addressed a different clinical target (tissue oxygenation/perfusion rather than infection classification), it was considered separately in the narrative synthesis and should be interpreted as a distinct exploratory application rather than as directly comparable evidence within the infection-detection subgroup.

Across the included studies, reporting of participant-level clinical and biological characteristics was frequently incomplete, with limited information on variables such as diabetes duration, glycaemic control, comorbidities, skin-related characteristics, and other potential modifiers of optical signal behaviour.

### 3.3. Devices and Procedures

Parekh et al. [27] provided the first clinical report on a handheld autofluorescence-based device later commercialised as Illuminate^®^, which was also used in subsequent studies by Kesavan et al. [28], Viswanathan et al. [29], and Rajendran et al. [30].

In four of the five studies, ulcer assessment was performed under controlled lighting conditions, either in a dark room or using a black hood, with non-contact image acquisition at distances ranging from 7–10 cm [27] to 10–12 cm [28] and acquisition times of approximately 20–30 s [27,28,29]. Krishnamoorthy et al. [31] did not report comparable acquisition details.

Different excitation strategies were adopted across studies, including ultraviolet and blue-light sources (400–500 nm) to excite endogenous and bacterial fluorophores (e.g., NADH, flavins, porphyrins, pyoverdine) [27] and multiple excitation wavelengths (370, 395, and 415 nm) for multispectral autofluorescence imaging [29]. Typically, more than 15 images per wound were acquired within 20–30 s [28,29].

Image analysis workflows relied on manual delineation of the region of interest on white-light images, followed by motion correction, thresholding, and spectral analysis to generate colour-coded wound maps indicating regions suggestive of Gram-positive or Gram-negative infection or absence of infection [28,29]. Kesavan et al. reported system calibration for detecting bacterial loads above $10^{4}$ CFU/g, considered clinically relevant [28].

Across these studies, the system also provided automated wound measurements (e.g., length, width, and occasionally area) alongside fluorescence outputs [27,28,29,30]. However, none of the articles described the underlying computation methods or reported dedicated validation of measurement accuracy. Taken together, the wound sizing functionality appears to be an available feature but remains insufficiently validated. The final output consists of a wound map with a colour overlay, where regions suggestive of Gram-positive bacteria are encoded in red, and those suggestive of Gram-negative bacteria in green [27].

A different handheld prototype (Illuminate-OxyView) was used by Krishnamoorthy et al. [31] to estimate tissue oxygen saturation ($StO_{2}$) in patients with DFU. In particular, the main aim described by the authors was to validate a machine learning approach applied to near-infrared (660, 740, and 850 nm) and co-registered white-light images. This study focused on pixel-wise $StO_{2}$ estimation and incorporated skin phototype modelling based on the Fitzpatrick scale, but provided limited information regarding the reference device used for comparison. A central contribution of the study was the explicit modelling of skin phototype (Fitzpatrick scale) to account for melanin-related effects on NIR reflectance measurements [31].

In all studies, device outputs were compared with reference standards, most commonly conventional microbiological culture [27,28,29,30], with one study also including 16S rRNA analysis for enhanced validation [28]. Krishnamoorthy et al. [31] instead used an FDA-cleared $StO_{2}$ imager as the reference standard, without further methodological details.

### 3.4. Artificial Intelligence Methods

A notable feature of the included literature is that most AI components were embedded within proprietary imaging platforms and reported with limited technical transparency; therefore, the review primarily captures device-integrated algorithmic applications rather than a broad spectrum of openly described AI methodologies.

Among the studies included in this scoping review [27,28,29,30], Krishnamoorthy et al. [31] provide the highest level of methodological clarity. Although code and data are not publicly available, the methodology is described in sufficient detail to enable conceptual replication. The extracted features are explicitly defined, the model class (Random Forest regressor) is clearly specified, and key elements such as hyperparameters, data splitting strategy, and evaluation protocol are reported. However, some limitations remain: the dataset composition and the performance of the segmentation model (U-Net + DenseNet-201) are not fully described, and the pixel-level formulation may limit insight into the distribution of patients across Fitzpatrick skin types.

In contrast, the other reviewed studies present substantial shortcomings in the description of the AI solutions employed. Viswanathan et al. [29] primarily emphasise the clinical implications of the device for patient management, rather than providing a necessary description of the underlying AI model. The authors refer to a “pretrained machine learning algorithm” that compares relative fluorophore intensities to assign one of three labels (Gram-positive, Gram-negative, or no infection) to image regions, generating a chromatic overlay on clinical photographs. However, neither the model type nor its architecture is specified, and no details are provided regarding the training dataset, training procedure, or validation scheme, precluding independent reproduction or auditing of the model.

A similar approach was previously adopted by Parekh et al. [27]. In this study, multispectral images were processed by an internal algorithm described as an “advanced computational algorithm” and a “proprietary state-of-the-art AI engine,” which integrates image-processing and machine learning techniques to detect clinically relevant bacterial burden. Despite this functional description, the technical details of the AI component remain highly abstract, and the model is effectively treated as a black box. Overall, the manuscript does not provide enough elements to enable independent reproduction or auditing of the proposed model.

Additional limitations concern the validation of ancillary device functionalities. Both Parekh et al. [27] and Kesavan et al. [28] report automated wound measurement features, including wound length and width estimation, displayed alongside fluorescence images and classification outputs. However, no dedicated experiments or validation tests are presented to assess the accuracy of these measurements [27,28]. Consequently, although wound size estimation is available within the system, its quantitative reliability remains unclear [27].

Relative to these studies, Kesavan et al. [28] represent a partial improvement in the reporting of AI methodology. The authors explicitly state that features extracted from multispectral images are provided as input to a Random Forest classifier, which outputs a four-class prediction (Gram-positive, Gram-negative, polymicrobial infection, or no clinically significant bacterial burden). Key aspects of the pipeline are reported, including the model type, the training and validation scheme, and the relationship between the device signal threshold and the microbiological threshold ($10^{4}$ CFU/g). Nonetheless, important details—such as source code, model parameters, and formal definitions of the extracted features—are not disclosed. As a result, while the pipeline can be reconstructed at a conceptual level, full implementation-level reproducibility is not achievable.

Even in the case of Rajendran et al. [30], the limited methodological description reported in the article is consistent with that present in other studies [27,28,29]. Despite being an additional study employing the same Illuminate device, no further technical details are provided regarding the embedded AI model or the training dataset used. The device is used as a pretrained instrument, and the AI component is again treated as a black box, without substantive new information beyond what was already available from prior studies.

Across the included studies, key AI-reporting elements were frequently missing, including model architecture, feature definition, dataset composition, training procedures, hyperparameter selection, validation strategy, and external testing. This limited the possibility of performing a comparative technical synthesis and should itself be considered a relevant finding of the review.

### 3.5. Outcome Estimation

Since four of the five included studies addressed broadly similar infection-related tasks, comparison of the reported outcome measures was possible at a descriptive level. Overall, performance estimates were often favourable within the respective study-specific evaluation settings. However, these results should be interpreted cautiously because they derive from small or homogeneous datasets, incompletely reported validation procedures, and a literature largely concentrated on closely related device platforms. As reported in Table 2, for the four works [27,28,29,30], the reported performance estimates were often favourable within the respective study-specific evaluation settings. Accuracy was defined as the proportion of correctly classified cases among all observations. Sensitivity and specificity represented the model’s ability to correctly identify positive and negative cases, respectively, while positive predictive value (PPV) and negative predictive value (NPV) indicated the probability that cases predicted as positive or negative were truly positive or negative.

As shown in Table 2, Parekh et al. [27] reported high performance in distinguishing bacterial growth from no growth, with sensitivity exceeding 99% and overall Gram-type diagnostic accuracy of 93.2%, showing strong agreement with culture results (Cohen’s κ = 0.86; Cramer’s V = 0.765). Kesavan et al. [28] evaluated Gram-negative, Gram-positive, polymicrobial, and no clinically significant bacterial burden classifications, reporting accuracies ranging from 83.6% to 94.9% and substantial agreement with culture (Cramer’s V = 0.774), although sensitivity was lower for polymicrobial and low-burden cases. Viswanathan et al. [29] assessed inference on new patients, achieving class-wise accuracies between 86.3% and 95.4%, a mean overall accuracy of 89.5%, and a multiclass AUC of 0.86, suggesting promising performance within the reported evaluation setting, although the available information is insufficient to establish robust generalisability. In contrast, Rajendran et al. [30], using a smaller dataset, reported lower accuracy for bacterial growth detection (71.2%) and moderate agreement with culture (κ = 0.659) and did not validate wound dimensional measurements.

Regarding the study of Krishnamoorthy et al. [31], the authors trained a Random Forest regressor at the pixel level, using a 70/30 train–test split, to estimate tissue oxygen saturation ($StO_{2}$) from combined NIR/RGB features. When trained on the pooled dataset including all skin phototypes, the model showed a strong agreement with the reference FDA-approved imager (Pearson r ≈ 0.97; RMSE ≈ 8.95%). To assess performance across skin tones, additional models were trained and evaluated on phototype-specific subsets (Fitzpatrick 3–6), achieving similarly high correlations (r = 0.93–0.97) with lower error for lighter phototypes and higher RMSE for darker skin tones. Qualitative results indicated that phototype-specific models reduced $StO_{2}$ overestimation observed with the generic model in Fitzpatrick 6 subjects, suggesting that skin-colour-specific training may mitigate melanin-related bias. Although the device includes a 3D camera for wound dimension measurement, no quantitative evaluation of this functionality was reported.

### 3.6. Limitations of the Considered Studies

Overall, the studies included in this scoping review share several methodological and technical limitations, largely reflecting overlapping aims and the use of similar imaging devices. In general, the available evidence is based on relatively limited sample sizes and narrowly defined clinical or technical settings, which may restrict the generalisability of the findings. In addition, several studies provide incomplete reporting of participant characteristics, making it difficult to fully interpret the representativeness of the study populations and to assess the applicability of results across different patient subgroups [27].

Another major limitation concerns the description of the AI component employed [27]. In several studies, the embedded AI models are insufficiently described and are essentially treated as proprietary “black boxes”, limiting reproducibility, independent validation, and assessment of algorithmic bias [27]. More broadly, inadequate transparency regarding model development, input features, training procedures, and performance assessment reduces the interpretability of the reported findings. This issue is further compounded by the lack of external validation, as most studies appear to rely on internally generated data only, with limited evidence on model robustness across independent cohorts, centres, or acquisition conditions.

Regarding the devices used in the five studies considered, it is important to note that the fluorescence-based systems require controlled lighting conditions and the use of a dark environment (dark room or “black hood”) [27]. Moreover, the devices can be susceptible to interference from tissue autofluorescence (largely collagen-related; e.g., bone, tendon, adipose tissue) [27,28,29], cleansing agents (such as Betadine) [27,29], sutures, and wound dressings, which may lead to false or ambiguous signals [28]. Therefore, thorough wound cleansing with saline before imaging is required [28,29].

The limited penetration depth (approximately 0.5–1 mm) restricts assessment to superficial infections and precludes evaluation of closed wounds [29].

In the case of polymicrobial infections, the devices considered in this scoping review typically report only the predominant Gram signal, limiting species-level discrimination and accurate quantification of mixed infections [27,29]. Additional limitations include exclusion of images due to acquisition issues, incomplete reporting or validation of ancillary device capabilities (e.g., wound size measurement) [30], and potential bias related to skin pigmentation in near-infrared measurements [31]. In particular, the authors reported that $StO_{2}$ estimation from NIR reflectance is often confounded by melanin concentration, including the tendency to overestimate saturation in individuals with higher melanin levels [31].

Finally, the evidence base is characterised by a substantial overlap in device families and technological platforms across studies. This concentration of evidence on closely related systems may limit the breadth of conclusions that can be drawn and raises the possibility that the available literature reflects, at least in part, dependence on a restricted set of proprietary technologies rather than independent validation across heterogeneous platforms.

## 4. Discussion

This scoping review indicates that interest in AI-assisted analysis of skin/wound autofluorescence signals for diabetic foot ulcer (DFU) assessment has emerged only recently. However, the available evidence remains limited. Only five studies [27,28,29,30,31], all conducted in India, met our inclusion criteria (Table 1). Four studies [27,28,29,30] evaluated the diagnostic performance of an autofluorescence-based device for detecting bacterial burden and classifying infection by Gram type in DFUs. Overall, these studies reported performance estimates that appear encouraging within restricted evaluation settings; however, these findings should be interpreted cautiously given the small number of studies, limited geographical diversity, incomplete reporting of AI validation procedures, and the absence of broad independent external validation.

Although a formal risk-of-bias tool was not applied, the included studies showed recurring methodological limitations that affect the interpretability of the reported findings. First, dataset size and diversity were often limited, and participant-level clinical descriptors were incompletely reported. This makes it difficult to assess representativeness, case-mix variability, and the extent to which the reported results may generalise beyond the original study samples. Second, validation was largely restricted to internal or study-specific settings, with no broad external validation across independent cohorts, centres, operators, or acquisition environments. Under these conditions, favourable performance estimates should be interpreted primarily as indicators of analytical feasibility rather than robust evidence of clinical validity or routine-care utility. Third, incomplete reporting of training procedures, model inputs, and evaluation design reduces the ability to assess potential overfitting and limits reproducibility and independent scrutiny.

The limited reporting of clinically relevant participant and ulcer descriptors, including comorbidities, ulcer severity or chronicity, treatment status, and skin-related variables, substantially reduces the interpretability and transferability of the reported diagnostic results. Moreover, four of the five studies evaluated the same device family (Illuminate or related variants), indicating that the available literature is not only small but also technologically narrow. Additionally, the current evidence base is heavily concentrated on infection-related applications, particularly bacterial burden detection and Gram-type classification; therefore, the review should not be interpreted as evidence of mature coverage across the broader spectrum of DFU assessment tasks, but as a mapping of early device-focused evidence.

A key methodological consideration is dataset size and diversity. AI models typically require sufficiently large and heterogeneous datasets to support robust parameter estimation, reduce overfitting, capture biological variability, and achieve reliable generalisation across clinical settings. Therefore, small-sample studies (e.g., Rajendran et al. [30]) should be considered preliminary and interpreted cautiously, as limited sample size may inflate performance estimates, increase uncertainty, and reduce external validity (Table 1 and Table 3).

From a clinical perspective, infection is a common and consequential complication of DFUs. Diabetic foot infections can delay or prevent wound healing and are associated with increased morbidity, mortality, healthcare utilisation, and risk of lower-limb amputation [32,33,34,35,36]. Estimates suggest that infection occurs in a substantial proportion of DFUs (approximately 60%) and contributes materially to adverse outcomes [34,35,36]. In this context, non-invasive and near-real-time tools that can support earlier detection, triage, and treatment monitoring could offer meaningful clinical value.

At the same time, DFU-related infections present with heterogeneous severity, ranging from superficial soft-tissue involvement to necrotising fasciitis, sepsis, and osteomyelitis. Osteomyelitis is common and is associated with an increased risk of amputation [37,38,39]. While debridement is required in many infected ulcers, amputation may still be necessary in a subset of cases (approximately 15–20%) [40]. Timely recognition and appropriate management are therefore critical; nevertheless, bacterial profiling can be resource-intensive and time-consuming, potentially delaying targeted interventions. Reports that a proportion of patients show evidence of infection even 10–20 days after debridement further underscore the need for reliable monitoring strategies [4].

Autofluorescence-based assessment may help address some of these needs, but practical and technical constraints must be considered. Signal interpretation may be influenced by multiple biological and technical confounders; however, the extent to which these factors were directly assessed varied across the included studies. Controlled lighting conditions and standardised image acquisition procedures were explicitly required in the infection-focused studies, indicating a recognised dependence on acquisition context. In the tissue oxygenation study, skin phototype and melanin-related bias were directly examined. By contrast, other plausible confounders, such as necrotic tissue, blood, exudate, or wound microenvironment heterogeneity, were not systematically evaluated across the included studies and therefore remain insufficiently characterised in relation to model performance.

In contrast to the infection-focused studies, Krishnamoorthy et al. [31] primarily described the Illuminate OxyView system and evaluated its performance for tissue oxygenation assessment, including the effect of skin type and melanin (Table 1). Although preliminary, their results were generally encouraging and potentially useful in patient care. In fact, tissue oxygen saturation ($StO_{2}$) assessment can be clinically relevant in DFUs for evaluating perfusion, estimating healing potential, and monitoring response to treatment.

Despite these promising signals, our synthesis highlights substantial limitations in the current literature. Participant characteristics were insufficiently reported in four of the five included studies, raising concerns regarding selection bias and limiting the interpretability and generalisability of the findings [23,26]. Another key finding of this review is the marked concentration of the evidence base within a single national context and, to a large extent, within one technological line. Four of the five included studies evaluated the same device family or closely related variants, and all studies were conducted in India. This pattern limits external validity at two levels: population-level generalisability and platform-level transferability. More specifically, the available literature may reflect progressive evaluation of a restricted proprietary ecosystem rather than independent confirmation of the broader clinical utility of AI-assisted autofluorescence across heterogeneous settings, operators, wound types, and technical platforms. This concentration also raises the possibility of platform-driven evidence, whereby the apparent maturity of the field may be shaped more by repeated study of the same technological solution than by true methodological diversification. In addition, reporting of the AI component was frequently inadequate. In biomedical publications, AI interventions should be described with sufficient detail regarding their role in the workflow, model type/configuration, training data, validation procedures, and intended use, to support transparency, reproducibility, clinical evaluability, and ethical oversight [41,42]. In the reviewed studies, the embedded AI models were often treated as proprietary black boxes, and key technical details (e.g., architecture, training dataset composition, and validation scheme) were missing [27,28,29,30,31]. These gaps hinder independent verification, bias assessment, and translation into broader clinical practice.

In summary, the available evidence suggests that AI-assisted autofluorescence approaches for DFU assessment are feasible and may provide clinically useful information, particularly for infection-related tasks. However, the field is still at an early stage. Larger, multicentre studies with diverse populations, standardised acquisition protocols, and transparent reporting of AI methodology are needed to establish clinical utility, robustness across settings, and the conditions under which these tools can be reliably integrated into routine DFU care.

Future studies in this area would benefit from adherence to structured AI-reporting frameworks, such as CLAIM for medical imaging studies and TRIPOD-AI for prediction model reporting, in order to improve reproducibility, auditability, and clinical evaluability.

## 5. Conclusions

Autofluorescence-based approaches have been increasingly explored in the management of diabetic foot ulcers (DFUs); however, only a small number of studies have combined autofluorescence assessment with artificial intelligence (AI) methods. The currently available evidence remains preliminary, geographically restricted, and technologically concentrated; therefore, it is insufficient to support broad conclusions regarding the overall clinical utility of AI-assisted autofluorescence in DFU care.

Overall, non-invasive autofluorescence-based tools may have potential relevance for DFU assessment and monitoring; however, the currently available studies do not yet provide sufficient evidence to establish cost-effectiveness, ease of integration into routine workflows, or broad clinical utility. In conclusion, at present, the available literature supports early proof-of-feasibility and analytical promise in restricted settings rather than robust evidence of broad clinical validity, implementation readiness, or real-world effectiveness.

## Figures and Tables

**Table 1:** Selected studies.

Study	Year	Aim	Device	Country	Sample Size	M/F Ratio	Reference Standard
[27]	2022	To evaluate the diagnostic accuracy of a novel handheld multispectral autofluorescence device for detecting bacterial infection and determining its Gram type in diabetic foot ulcers, compared with standard tissue culture	Illuminate	India	100	88/12	Microbiological culture
[28]	2022	To evaluate the diagnostic accuracy of an AI-enabled multispectral autofluorescence imaging device for determining bacterial Gram type in diabetic foot ulcers, compared with standard deep-tissue culture methods	Illuminate	India	157	104/53	Microbiological culture
[29]	2024	To assess the clinical diagnostic accuracy of an AI-enabled autofluorescence imaging device for Gram-type classification in diabetic foot ulcers	Illuminate	India	178	144/34	Microbiological culture
[30]	2025	To evaluate the diagnostic performance of a multispectral autofluorescence imaging device for detecting bacterial burden and Gram type in diabetic foot ulcers, using standard wound swab culture as the microbiological reference	Illuminate	India	15	10/5	Microbiological culture
[31]	2025	To build and validate an AI model that predicts $StO_{2}$ maps in foot ulcers from RGB and NIR imaging	Illuminate OxyView	India	300	nr	$StO_{2}$ imager

nr: not reported in the study.

**Table 2:** Performance of AI models. For multiclass problems, values for sensitivity, specificity, PPV, and NPV are reported as ranges (min–max) across the reported classes.

Study	Accuracy (%)	Sensitivity (%)	Specificity (%)	PPV (%)	NPV (%)	AUC	r	RMSE	Cohen’s κ	Cramer’s V
[27]	93.2	99.24	82.35	97.76	93.33	–	–	–	0.86	0.765
[28]	83.6–94.9	57.1–94.6	71.8–98.7	76.0–92.3	88.1–97.4	–	–	–	0.774	–
[29]	89.5	77.2–91.3	82.1–98.4	80.8–91.7	87.3–96.1	0.86	–	–	–	–
[30]	71.2–84.2	85.7–92.3	50.0–83.3	85.7–92.3	50–83.3	–	–	–	0.659	0.688
[31]	–	–	–	–	–	–	0.93–0.97	4.33–10.3	–	–

**Table 3:** Comparative overview of AI methods. “—” means that the information is not clearly reported in the paper.

Study	AI Model	AI Input	Ground Truth	Dataset	Train Strategy	Evaluation Metrics
[27]	ML classifier	—	Deep-tissue biopsy culture	149 wound biopsies	—	SS, SP, PPV, NPV, AC, κ, *V*
[28]	RF classifier	Spectral intensity features	Deep-tissue biopsy culture	177 wound biopsies	80% train, 20% test	SS, SP, PPV, NPV, AC, κ
[29]	ML classifier	—	Deep-tissue biopsy culture	203 wound biopsies	—	SS, SP, PPV, NPV, AC, AUC
[30]	AI classifier	—	Wound swab culture	15 wound swabs	—	SS, SP, PPV, NPV, AC, κ, *V*
[31]	RF classifier	21 per-pixel features	FDA-cleared $StO_{2}$ imaging device	300 patients	70% train, 30% test	*r*, RMSE

SS: sensitivity; SP: specificity; PPV: positive predictive value; NPV: negative prediction value; AC: accuracy; κ: Cohen’s kappa value; *V*: Cramer’s V; AUC: multiclass area under the ROC curve; *r*: Pearson correlation coefficient; RMSE: root mean square error.

## Data Availability

No new data were created or analysed in this study. Data sharing is not applicable to this article.

## Supplementary Materials

The following supporting information can be downloaded at: https://www.mdpi.com/article/10.3390/jpm16060285/s1, Supplementary Table S1: PRISMA checklist.

## Appendix A. Search Strings for Each Database

### Appendix A.1. Scopus

**Search string (as executed):** *TITLE-ABS-KEY ((“Diabetic foot” OR “Diabetic foot ulcer” OR “lower extremity ulcer” OR “wound healing” OR “neuropathic foot” OR “ischemic foot” OR “diabetic foot syndrome”) AND (“autofluoresce” OR “autofluoresced” OR “autofluorescence” OR “autofluorescent” OR “autofluorescing” OR “fluoresce” OR “fluoresced” OR “fluorescence” OR “fluorescences” OR “fluorescent” OR “fluorescently” OR “fluoresces” OR “fluorescing” OR “fluorescence imaging” OR “tissue fluorescence” OR “intrinsic fluorescence” OR “skin fluorescence”) AND (“artificial intelligence” OR “AI” OR “deep learning” OR “neural network” OR “machine learning” OR “genetic algorithm” OR “predictive models” OR “data mining”)) AND PUBYEAR > 2009 AND PUBYEAR < 2027 AND (LIMIT-TO (DOCTYPE,“ar”) OR LIMIT-TO (DOCTYPE,“cp”)) AND (LIMIT-TO (LANGUAGE,“English”)) AND (EXCLUDE (EXACTKEYWORD,“Mouse”) OR EXCLUDE (EXACTKEYWORD,“Nonhuman”) OR EXCLUDE (EXACTKEYWORD,“Animal”) OR EXCLUDE (EXACTKEYWORD,“Animals”) OR EXCLUDE (EXACTKEYWORD,“Animal Experiment”)).*

### Appendix A.2. Pubmed

**Search string (as executed):** *(“Diabetic foot” OR “Diabetic foot ulcer” OR “lower extremity ulcer” OR “wound healing” OR “neuropathic foot” OR “ischemic foot” OR “diabetic foot syndrome”) AND (“autofluoresce” OR “autofluoresced” OR “autofluorescence” OR “autofluorescent” OR “autofluorescing” OR “fluoresce” OR “fluoresced” OR “fluorescence” OR “fluorescences” OR “fluorescent” OR “fluorescently” OR “fluoresces” OR “fluorescing” OR “fluorescence imaging” OR “tissue fluorescence” OR “intrinsic fluorescence” OR “skin fluorescence”) AND (“artificial intelligence” OR “AI” OR “deep learning” OR “neural network” OR “machine learning” OR “genetic algorithm” OR “predictive models” OR “data mining”).*

Additional filters:

- **Publication date:** from 2010 to 2026;
- **Article language:** English;
- **Species:** Humans.

### Appendix A.3. Web of Sciences

**Search string (as executed):** *(“Diabetic foot” OR “Diabetic foot ulcer” OR “lower extremity ulcer” OR “wound healing” OR “neuropathic foot” OR “ischemic foot” OR “diabetic foot syndrome”) AND (“autofluoresce” OR “autofluoresced” OR “autofluorescence” OR “autofluorescent” OR “autofluorescing” OR “fluoresce” OR “fluoresced” OR “fluorescence” OR “fluorescences” OR “fluorescent” OR “fluorescently” OR “fluoresces” OR “fluorescing” OR “fluorescence imaging” OR “tissue fluorescence” OR “intrinsic fluorescence” OR “skin fluorescence”) AND (“artificial intelligence” OR “AI” OR “deep learning” OR “neural network” OR “machine learning” OR “genetic algorithm” OR “predictive models” OR “data mining”).*

Additional filters:

- **Document types:** Article or Proceeding Paper;
- **Languages:** English.

### Appendix A.4. Embase

**Search string (as executed):** *(‘diabetic foot’:ti,ab OR ‘diabetic foot ulcer’:ti,ab OR ‘lower extremity ulcer’:ti,ab OR ‘wound healing’:ti,ab OR ‘neuropathic foot’:ti,ab OR ‘ischemic foot’:ti,ab OR ‘diabetic foot syndrome’:ti,ab) AND (‘autofluoresce’:ti,ab OR ‘autofluoresced’:ti,ab OR ‘autofluorescence’:ti,ab OR ‘autofluorescent’:ti,ab OR ‘autofluorescing’:ti,ab OR ‘fluoresce’:ti,ab OR ‘fluoresced’:ti,ab OR ‘fluorescence’:ti,ab OR ‘fluorescences’:ti,ab OR ‘fluorescent’:ti,ab OR ‘fluorescently’:ti,ab OR ‘fluoresces’:ti,ab OR ‘fluorescing’:ti,ab OR ‘fluorescence imaging’:ti,ab OR ‘tissue fluorescence’:ti,ab OR ‘intrinsic fluorescence’:ti,ab OR ‘skin fluorescence’:ti,ab) AND (‘artificial intelligence’:ti,ab OR ‘ai’:ti,ab OR ‘deep learning’:ti,ab OR ‘neural network’:ti,ab OR ‘machine learning’:ti,ab OR ‘genetic algorithm’:ti,ab OR ‘predictive models’:ti,ab OR ‘data mining’:ti,ab).*

Additional filters:

- **Publication date:** from 2010 to 2025;
- **Text availability:** Article;
- **Study type:** cell culture technique OR clinical article OR clinical study OR comparative study OR controlled study OR diagnostic test accuracy study OR human OR human cell OR in vitro study OR major clinical study OR physical model OR pilot study OR random walk OR randomized controlled trial OR simulation OR tissue culture.

### Appendix A.5. CinaHL

**Search string (as executed):** *(“Diabetic foot” OR “Diabetic foot ulcer” OR “lower extremity ulcer” OR “wound healing” OR “neuropathic foot” OR “ischemic foot” OR “diabetic foot syndrome”) AND (“autofluoresce” OR “autofluoresced” OR “autofluorescence” OR “autofluorescent” OR “autofluorescing” OR “fluoresce” OR “fluoresced” OR “fluorescence” OR “fluorescences” OR “fluorescent” OR “fluorescently” OR “fluoresces” OR “fluorescing” OR “fluorescence imaging” OR “tissue fluorescence” OR “intrinsic fluorescence” OR “skin fluorescence”) AND (“artificial intelligence” OR “AI” OR “deep learning” OR “neural network” OR “machine learning” OR “genetic algorithm” OR “predictive models” OR “data mining”).*

Additional filters:

- **Publication date:** from 1 January 2010 to 31 December 2026;
- **Languages:** English;
- **Species:** Humans.

### Appendix A.6. SPIE Digital Library

**Search string (as executed):** *((“Diabetic foot” OR “Diabetic foot ulcer” OR “lower extremity ulcer” OR “wound healing” OR “neuropathic foot” OR “ischemic foot” OR “diabetic foot syndrome”)) AND ((“autofluorescence” OR “autofluorescent” OR “fluorescence” OR “fluorescence imaging” OR “tissue fluorescence” OR “intrinsic fluorescence” OR “skin fluorescence”)) AND ((“artificial intelligence” OR “AI” OR “deep learning” OR “neural network” OR “machine learning” OR “genetic algorithm” OR “predictive models” OR “data mining”)) NOT ((Mouse OR Nonhuman OR Animal OR Animals OR “Animal Experiment”)).*

Additional filters:

- **Publication type:** Conference Proceedings, Paper and Journal Article;
- **Publication date:** from 2010 to 2026.

### Appendix A.7. Google Scholar

**Search string (as executed):** *(“Diabetic foot” OR “Diabetic foot ulcer” OR “lower extremity ulcer” OR “wound healing” OR “neuropathic foot” OR “ischemic foot” OR “diabetic foot syndrome”) AND (“autofluoresce” OR “autofluoresced” OR “autofluorescence” OR “autofluorescent” OR “autofluorescing” OR “fluoresce” OR “fluoresced” OR “fluorescence” OR “fluorescences” OR “fluorescent” OR “fluorescently” OR “fluoresces” OR “fluorescing” OR “fluorescence imaging” OR “tissue fluorescence” OR “intrinsic fluorescence” OR “skin fluorescence”) AND (“artificial intelligence” OR “AI” OR “deep learning” OR “neural network” OR “machine learning” OR “genetic algorithm” OR “predictive models” OR “data mining”).*

Additional filters:

- **Where words occur** in the title of the article;
- **Publication date:** from 2010 to 2026.
